# Tripping into the unknown: Exploring the experiences of first-time LSD users through global drug survey insights

**DOI:** 10.1177/02698811241254837

**Published:** 2024-05-28

**Authors:** Luke Baxter, Cheneal Puljević, Tim Piatkowski, Jason Ferris, Emma L Davies, Monica J Barratt, Adam Winstock

**Affiliations:** 1 South London and Maudsley NHS Trust/King’s College London, London, UK; 2 School of Public Health, The University of Queensland, Brisbane, QLD, Australia; 3 School of Applied Psychology, Griffith Centre for Mental Health, Griffith University, Mount Gravatt, QLD, Australia; 4 Centre for Health Services Research, The University of Queensland, Brisbane, QLD, Australia; 5 Centre for Psychological Research, Oxford Brookes University, Oxford, UK; 6 Social Equity Research Centre and Digital Ethnography Research Centre, RMIT University, Melbourne, VIC, Australia; 7 National Drug and Alcohol Research Centre, UNSW Sydney, Sydney, NSW, Australia; 8 Global Drug Survey, London, UK; 9 Institute of Epidemiology, University College London, London, UK

**Keywords:** Drugs, harm reduction, LSD, peers, psychedelic

## Abstract

**Background:**

The recreational use of LSD, a synthetic psychedelic drug, has surged in recent years, coinciding with a renewed research focus on its potential psychotherapeutic properties.

**Aim:**

This study aims to describe the experiences and perceptions of individuals engaging in LSD use for the first time, derived from a large international sample.

**Methods:**

This study utilised 2018 Global Drug Survey data collected from 6 November 2017 to 10 January 2018. Participants who initiated LSD use in the preceding 12 months answered questions on their experiences, social settings, harm-reduction behaviours, and demographics. Descriptive statistics were employed, and characteristics of those seeking emergency medical treatment (EMT) and those not planning further LSD use were compared with other respondents.

**Results:**

Among 3340 respondents who used LSD in the past year, their first-time experiences generally exceeded expectations, with 97.7% expressing excitement. Adverse and unwanted side effects were rarely reported, and only 17 individuals needed EMT. Feelings of fear were reported by most (64.1%), but only very mildly and not enough to put them off from wanting to use LSD again.

**Discussion:**

Although the occurrence of unwanted side effects seems low and the LSD experience is generally pleasurable, vigilance amid the rising illicit use of LSD through harm-reduction education is still important in preventing possible risks.

## Introduction

Lysergic Acid Diethylamide (LSD) is a synthetic psychedelic hallucinogen, first discovered in 1943. Despite an early role in psychiatric research, by the mid-1960s, its recreational use had become widespread, leading to its categorisation as a Schedule I substance during 1971 United Nations Conventions on Drugs (Khan, 1979; Nichols and Walter, 2021). In recent years, there has been a notable rise in the recreational usage of LSD, witnessing a substantial increase of 200% from the early 2000s to the late 2010s (Killion et al., 2021; Yockey et al., 2020). There has also been renewed research interest in LSD and psychedelics, more generally, as therapeutic agents (Carhart-Harris and Goodwin, 2017; Fuentes et al., 2020; Ko et al., 2023). Additionally, the practice of LSD ‘microdosing’, in which sub-hallucinogenic doses of the drug are taken regularly with the aim of improving cognitive or social functioning, is gaining in popularity (Cameron et al., 2020). As a result, it is pertinent to consider, more fully, the attitudes and behaviour of new LSD consumers, particularly in the context of public health harm-reduction strategies outside the remit of psychedelic-assisted treatment, amidst rising LSD use trends.

LSD is a prototypical classic hallucinogen that exerts its function by modulating the serotonin system, leading to profound alterations in perception, cognition, and sensory experiences (Carhart-Harris et al., 2016). It presents a relatively low risk in terms of its physiological adverse effects, danger of toxicity and overdose, likelihood of dependence, and overall social harm when compared to other recreational drugs (Schlag et al., 2022). Nevertheless, under the influence, people who use LSD may have adverse experiences, colloquially known as ‘bad trips’, that are characterised by paranoia, anxiety, and confusion. While these effects tend to be transient (Carhart-Harris et al., 2016; Schmid et al., 2015), in some cases perceptual disturbances from the use of psychedelics may persist (Martinotti et al., 2018).

Challenging experiences can be influenced by the social and cultural environment in which LSD is taken (the ‘setting’) and the psychological state of the individual at the time of taking (the ‘mindset’ or ‘set’): taking the drug in unfamiliar surroundings, while in a negative state of mind, with people who are unknown, untrusted or unfamiliar with the drug, is more likely to lead to a ‘bad trip’ (Palmer and Maynard, 2022). These experiences are rare, however. Data from the Global Drug Survey (GDS) 2017 show that just 1% of individuals who had used LSD in the previous year reported seeking emergency medical treatment (EMT) (Kopra et al., 2022). The most common symptoms requiring EMT that were described tended to be psychological rather than physical, and the most cited reasons for the adverse experience among participants were ‘wrong setting’ and ‘wrong mindset’ (Kopra et al., 2022). While first-time use was not found to be a predictor of EMT-seeking in this study, it may be contested whether this conclusion is definitive, given that the sample of EMT-seekers was small. Moreover, EMT-seekers tended to be younger, suggesting a lack of experience (Kopra et al., 2022). Indeed, the perception of ‘bad trips’ among psychedelic-use veterans is that they tend to be associated with novices, particularly those who have an ‘immature’ approach toward set and setting (Gashi et al., 2021). In keeping with this, people who take psychedelics report a greater use of harm-reduction methods, including attention paid to set and setting, for their most recent experience in comparison to their first experience (Palmer and Maynard, 2022).

Recent GDS results have indicated that many respondents have taken psychedelics to enhance their own wellbeing, to cope with worrying, or to treat mental health difficulties (Winstock et al., 2021). As evidence for the therapeutic uses of psychedelic substances continues to be published (Carhart-Harris and Goodwin, 2017; Fuentes et al., 2020; Ko et al., 2023) and with growing public interest in psychedelic therapy (Danias and Appel, 2023), it is conceivable that many people taking LSD for the first time may feel emboldened to try it in an attempt to self-treat pre-existing psychiatric conditions: Increased familiarity with psychedelic scientific literature has been associated with more positive attitudes to psychedelic treatment (Kim and Suzuki, 2023), and, among individuals who self-medicate, perceived ‘good knowledge’ about available treatments was cited as a justification for this behaviour (Gras et al., 2020). Moreover, the initiation of substance use due to self-treatment and enhancement has been documented among consumers of other illicit substances (Piatkowski et al., 2023a, 2023b), and surveys have identified that self-treatment of mental health issues is a common motivator for psychedelic use (Mason et al., 2018; Lea et al., 2020). Promotion of effective harm-reduction practices among self-treating LSD consumers is important given their vulnerabilities to adverse experiences – comorbid mental health conditions are associated with an increased risk of EMT-seeking after taking LSD (Kopra et al., 2022). Moreover, people using LSD for the first time may also be vulnerable to adverse effects due to their unfamiliarity with dosing – LSD can provoke intense experiences even when taken at doses in the range of micrograms and has a long duration of action (Okumuş et al., 2023). LSD also produces more intense subjective effects than are seen with other illicit drugs (Holze et al., 2020), which could render the psychedelic experience, for those who have underprepared for their first time using the drug, potentially overwhelming. For this reason, taking a small ‘test dose’ is recommended for people using LSD for the first time so that they can determine their sensitivity to its psychedelic effects in a controlled manner.

Understanding the attitudes, drug-taking behaviour, and information sources of people taking LSD for the first time holds significant implications for informing public health harm-reduction strategies, particularly amidst the escalating trends of LSD use. This paper sought to understand the motivations, planning, experiences, and perceptions of individuals engaging in LSD use for the first time, derived from a large international sample.

## Methods

### Design

The GDS is an annual, anonymous, and encrypted online survey on substance use. It is advertised on social networking sites in collaboration with media partners and harm-reduction organisations. Full details about the survey design and recruitment, including related discussion on the survey’s utility, can be found elsewhere (Barratt et al., 2017; Winstock et al., 2022). The results from the present study are drawn from the GDS 2018. Briefly, GDS 2018 data collection was conducted between 6 November 2017 and 10 January 2018 and was completed by 130,761 participants from 206 countries. GDS respondents are recruited through social media (e.g. Facebook and Twitter) and through global media partners, such as Mixmag, Fairfax Media and The Guardian. It was translated into 18 languages: English, German, Serbian, Czech, Georgian, Azerbaijani, Hebrew, Polish, French, Italian, Spanish (S. American Spanish), Portuguese, Flemish, Hungarian, Turkish, Finnish and Danish. The survey gathered data concerning substance use patterns, specifically targeting individuals who reported using LSD, MDMA or cocaine for the first time in the last 12 months.

### Procedure and materials

Ethical approval was received from University College London 11671/001: GDS, The University of New South Wales (HREC HC17769) and University of Queensland (No: 2017001452) Research Ethics Committees. The survey collected a comprehensive set of demographic data from the participants. Subsequent sections of the survey prompted respondents to specify the last time they used various drugs, including LSD, by selecting from predetermined time frames (never, in the last 30 days, between 31 days and 12 months ago, more than 12 months ago). Participants who indicated prior usage of a specific drug were then directed to sections containing detailed inquiries concerning their experiences with these substances.

Participants who reported previous use of LSD were further queried whether they had used it for the first time within the preceding 12 months. If they had, this group was asked if they had used LSD the most recently out of LSD, cocaine and MDMA, as this was the order of the survey. Respondents reporting most recent use of LSD were directed to a module of questions pertaining to this initial experience, including the factors influencing their decision to use, planning and knowledge before use, dose, source from which the drug was acquired, other substances taken at time of taking the drug, the environment in which the drug was taken, whether they were accompanied by others at the time of taking the drug, whether they had to seek emergency medical treatment (EMT), and whether they intended to use or had used again. Finally, participants were asked about what their expectations of LSD had been prior to taking it and were asked to rate how positive or negative they had found the experience.

Example questions include: Thinking about the first time you used LSD, where did you get it from? Thinking about the first time you used LSD, what best describes the situation you were in? Thinking about the first time you used LSD, were you given any advice about how much to take? Before you used LSD for the first time, what sources did you obtain this information from? A full list of the questions relevant to this paper is found in the appendix. Demographic information pertaining to age, gender identity, highest academic qualification and country of origin was also obtained for respondents.

### Data analysis

Descriptive statistics and visual representations were created to explore the circumstances and experiences of first-time use among respondents. When frequencies and percentages are given in the results section, these describe the number and percentage of respondents to individual items in the survey, not the survey overall.

To explore the characteristics of EMT-seekers and respondents who said that they did not plan to use LSD again after the first time, Chi-square statistics were used to establish whether there was any association between membership of these groups and questions pertaining to the setting in which LSD was taken, participants’ mindset prior to taking LSD, and knowledge about LSD. When expected cell numbers were low, Fisher’s Exact test was used. The questions used to characterise EMT-seekers and respondents not planning to take LSD again are listed in the appendix.

EMT-seekers and respondents who did not plan to use LSD again were also compared to other respondents on a series of questions that measured using a 10-point scale mood prior to taking LSD, expectations of positive and adverse effects of LSD, and ratings of the pleasurableness of the experience on LSD and severity. Comparisons between groups were carried out using Mann-Whitney U tests. All statistical analyses were carried out using IBM SPSS statistics version 29.0.1.0.

## Results

Three thousand three hundred forty respondents were directed to the special module of questions pertaining to first-time use of LSD after indicating they had used LSD for the first time in the previous 12 months. These respondents were included in this study’s demographic analyses. Respondents’ socio-demographic data are shown in Table 1.

**Table 1.** Demographics for people with recent first-time use of LSD.

Age	
Mean (SD)	21.7 (5.3)
Median (range, IQR)	20.0 (16–59, 18–24)
Gender identity	*N* (%)
Male	2578 (77.2)
Female	707 (21.2)
Non-binary	42 (1.3)
Other	13 (0.4)
Country of origin	*N* (%)
Germany	831 (24.9)
United States	516 (15.4)
Poland	330 (9.9)
United Kingdom	225 (6.7)
Denmark	206 (6.2)
Australia	140 (4.2)
Austria	84 (2.5)
Canada	80 (2.4)
Slovakia	79 (2.4)
Netherlands	75 (2.2)
Other	849 (25.4)
Total	3340 (100)

Participants who reported using LSD for the first time in the previous 12 months were also asked about their highest level of academic qualification. There were 2703 respondents who chose to answer this question; the data are shown in Table 2.

**Table 2.** Highest academic qualification of people taking LSD for the first time.

Highest academic qualification	*N* (%)
No formal schooling	10 (0.4)
Primary school	165 (6.1)
Lower secondary school	390 (14.4)
Technical or trade certificate	167 (6.2)
Higher secondary school	744 (27.5)
College certificate/diploma	590 (21.8)
Undergraduate degree	497 (18.4)
Postgraduate degree	109 (4.0)
Unsure/Don’t know	31 (1.1)
Total	2703 (100)

## First-time LSD consumption behaviours

### Timing

When asked about the timing of their first-time LSD use, participants who had used LSD for the first time in the previous 12 months provided responses indicating the amount of time that had elapsed between their first-time use and their completion of the survey. There were 3311 respondents who provided this information, which is illustrated in Table 3.

**Table 3.** Timing of first LSD consumption.

Timing	*N* (%)
In the last week	124 (3.7)
7–30 days ago	334 (10.1)
1–5 months ago	1444 (43.6)
6–12 months ago	1409 (42.6)
Total	3311 (100)

### Decision to take LSD

Participants were asked about their certainty regarding trying LSD before their first-time use. Among the 3240 respondents who provided this information, 57.0% stated that they ‘always knew they would take it 1 day’, indicating a majority had a strong prior inclination towards trying LSD.

When asked about the planning of their first-time LSD use, the majority (69.4%) of respondents (*n* = 3222) stated that their first experience with LSD was planned.

Regarding the influence of friends on the decision to try LSD, few participants provided responses indicative of strong peer influence. Among the 3220 respondents who answered this question, the majority (63.3%) stated that their friends’ influence was not strong at all.

Participants’ views on the importance of their friends’ support in the decision to try LSD were also obtained. The results suggest that the majority of participants did not assign great importance to their friends’ support in their decision to try LSD. Further information pertaining to the decision to take LSD is found in Table 4.

**Table 4.** Factors influencing decision to take LSD for the first time.

Before your first time, how certain were you that you would try LSD?	N (%)
I always knew I’d take it 1 day	1846 (57.0)
I thought I might take it at some point	1220 (37.7)
I have never considered using it before the day I took it for the first time	174 (5.4)
Total	3240 (100)
Thinking about the first time you used LSD was this first occasion	
Planned (you ensured you had access to LSD on that day)	2235 (69.4)
Spontaneous (LSD happened to be available on that day)	987 (30.6)
Total	3222 (100)
Thinking about the first time you used LSD, how strong was the influence of your friends in your decision to try it?	
Not strong at all	2037 (63.3)
Somewhat strong	858 (26.6)
Strong	228 (7.1)
Very strong	97 (3.0)
Total	3220 (100)
Thinking about the first time you used LSD, how important was it for you that your friends supported your decision to try it?	
Not important at all	1267 (39.3)
Somewhat important	1072 (33.3)
Important	625 (19.4)
Very important	256 (8.0)
Total	3220 (100)

### Acquisition, form and dosage of LSD

Respondents who had used LSD for the first time in the previous 12 months were asked about the source from which they obtained the drug. Among the 2451 participants who responded to this question, 30.4% reported getting LSD from their friends. Friends of friends were the source for 17.7% of participants, while 16.0% obtained LSD from dealers they knew personally. The findings indicate that friends and acquaintances are predominant suppliers of LSD for people using it for the first time, followed by personal dealers and darknet markets.

Participants were asked about the form of LSD that they took during their first time. Among the 3131 who answered this question, the majority, 80.6%, reported taking ‘blotters’.

Additional information on the acquisition source and form of LSD that was consumed is found in Table 5.

**Table 5.** Acquisition sources and forms of LSD taken for first time.

Source of LSD	*N* (%)
Friends	745 (30.4)
Friends of friends	435 (17.7)
Dealers that you know	392 (16.0)
Darknet markets (purchased by you directly)	333 (13.5)
Open websites (not darknet markets)	242 (9.9)
Darknet markets (purchased by someone else)	119 (4.9)
On the street/festival/club (dealers that you don’t know)	85 (3.5)
Other social media apps (e.g. Snapchat, Instagram, Tinder)	42 (1.7)
WhatsApp	15 (0.6)
Shopfronts (e.g. adult stores, head shops, coffee shops, smoke shops, cannabis shops)	5 (0.2)
Another source	38 (1.6)
Total	2451 (100)
Form of LSD	*N* (%)
Blotters	2523 (80.6)
Pills/tablets	193 (6.1)
Powder/crystal/paste	50 (1.6)
Multiple forms	15 (0.5)
Capsules	11 (0.4)
Other	339 (10.8)
Total	3131 (100)

**Figure 1.**
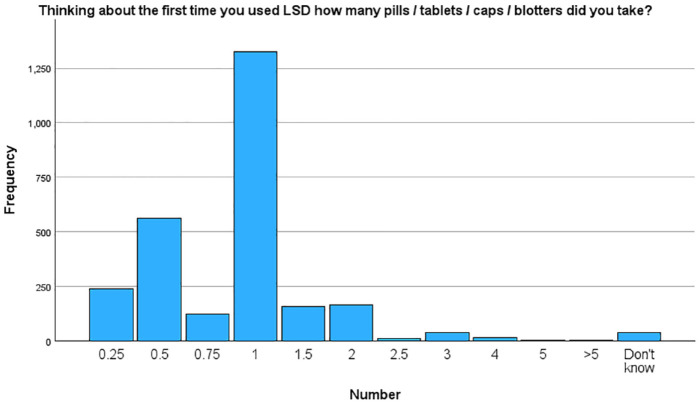
LSD dosages for first-time use, by dosing unit.

Participants were also asked about the dose of LSD that they consumed on their first time taking it, expressed in number of pills, tablets, caps or ‘blotters’ of LSD taken. There were 2684 respondents who answered this question. Their answers are illustrated in Figure 1.

### Setting and social accompaniment

Participants were asked to describe the situation in which they used LSD for the first time. Among the 3225 respondents who answered this question, the most common setting was at home, accounting for 30.3% of responses. Responses for other settings are shown in Table 6.

**Table 6.** Setting in which LSD was taken for the first time.

Setting	*N* (%)
At home	976 (30.3)
At a friend’s house	837 (26.0)
Outdoors/place of beauty	666 (20.7)
At a festival	280 (8.7)
At a private party	161 (5.0)
At an entertainment venue (club, pub, concert)	134 (4.2)
Other public space	97 (3.0)
At work	10 (0.3)
Online or virtual space (communicating with someone else or others)	3 (0.1)
Other place not described above	61 (1.9)
Total	3225 (100)
Thinking about the first time you used LSD, which option best describes who you were with?	
Close friend/group of friends	2232 (68.3)
On my own	449 (13.7)
With a partner/lover	368 (11.3)
Large group of people including strangers (e.g., at a club, festival)	207 (6.3)
With a medical professional/healer/guide	13 (0.4)
Total	3269 (100)
Thinking about the first time you used LSD, had the other people you were with used LSD before?	
Some of them had used it before	861 (31.0)
None of them had used it before	840 (30.2)
All of them had used it before	678 (24.4)
Most of them had used it before	380 (13.7)
Don’t know/unsure	18 (0.6)
Total	2777 (100)
Thinking about the first time you used LSD, was there a person whose primary role was to take care of you (e.g. trip-sitter/babysitter)?	
Yes	1372 (50.3)
No	1246 (45.7)
Don’t know/unsure	109 (4.0)
Total	2727 (100)

Respondents were asked to identify who they were with during their first LSD experience. Among the 3269 participants who responded to this question, the majority (68.3%) reported being with close friends or a group of friends.

Participants were asked about the LSD experience of the individuals they were with during their first-time LSD use. Among the 2777 respondents who provided this information, the responses provided suggest a diverse range of experiences among the individuals present during first-time LSD use.

Respondents were asked whether there was a person whose primary role was to take care of them during their first LSD experience (e.g. a ‘trip-sitter’ or ‘babysitter’). Among the 2727 participants who answered this question, 50.3% had someone in such a role. A summary of social setting arrangements is shown in Table 6.

### Disclosure

Participants who reported using LSD for the first time with other people were asked whether they informed at least one of the other people they were with that it was their first time using LSD. Among the 2754 respondents who answered this question, 91.6% indicated that they disclosed this information before the session started, 3.0% after the session started, and 0.6% after the session ended. Interestingly, 1.1% claimed to others to have used LSD before, while a very small proportion (0.8%) were unsure whether they had disclosed their first-time use to others.

### Mindset prior to taking LSD

**Figure 2.**
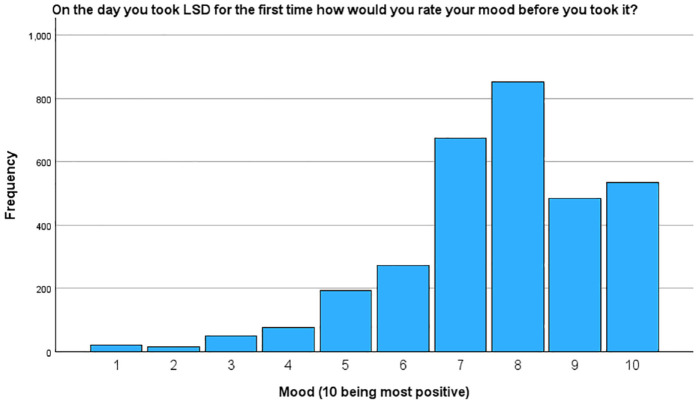
Participant’s mood before taking LSD for the first time.

Participants were asked to rate their mood prior to taking LSD for the first time on a scale of 1–10, with 10 being the most positive. Among the 3176 respondents who provided this information, the mean score was 7.7, with a standard deviation of 1.8. This indicates that, on average, participants reported a relatively positive mood before their first-time LSD experience. This is illustrated in Figure 2.

Regarding feelings of fear about taking LSD for the first time, respondents provided mixed responses. Among the 3183 participants who answered this question, 35.9% stated that they were not scared at all, 60.0% felt a little scared, and 4.1% felt very scared. The results show that a majority of participants had some level of fear, ranging from mild to significant, about using LSD for the first time.

In contrast, participants’ excitement about taking LSD was also explored. Among the 3180 respondents who answered this question, 2.3% reported not feeling excited at all, 29.8% felt a little excited, and a substantial 67.9% felt very excited. The findings indicate that the majority of participants had a high level of excitement about their upcoming first-time LSD experience.

### Polysubstance use

Participants were asked if they had taken any other drug on the day they used LSD for the first time before they took LSD. Among the 3225 respondents who answered this question, Table 7 illustrates the prevalence of polysubstance use. The data show that 38.8% of participants reported using only LSD on the day of their first-time experience. On the other hand, 59.6% of participants indicated using one or more additional substances in combination with LSD. A small proportion of respondents, 1.6%, could not remember if they had used any additional substances. In response to the question, ‘On the day that you used LSD for the first time, did you also try another drug/s for the first time?’ (*n* = 3135), 96.3% of participants stated that they did not try any other drug for the first time on that day. A small proportion of respondents, 3.6%, mentioned that they did try another drug for the first time on the same day as their first LSD use. A very minor percentage, 0.1%, could not remember whether they had tried another drug for the first time on that day.

**Table 7.** Participants reporting on any other drug use on the day they used LSD for the first time.

Drug	*N* (%)^a^
Cannabis	1459 (45.2)
Alcohol	1056 (32.7)
Ecstasy/MDMA	474 (14.7)
Amphetamine/methamphetamine	259 (8.0)
Cocaine	212 (6.6)
Other drugs not listed	453 (14.0)
Can’t remember/don’t know	50 (1.6)
None	1252 (38.8)

a Participants could select more than one option, hence percentages totalling greater than 100.

## Knowledge and expectations of LSD

Participants were asked about their knowledge of LSD, sources of information about the drug, and their expectations and experiences related to its effects and risks.

### Knowledge about LSD

When asked about their knowledge of the drug, its effects, and its risks before their first-time use, respondents (*n* = 3190) provided various responses. Almost half, 49.2%, reported having ‘a lot’ of knowledge about LSD, while 37.3% claimed to know ‘quite a bit’. A smaller percentage, 12.6%, indicated having ‘a little’ knowledge. Only 0.9% reported knowing ‘nothing’ about the drug before taking it.

### Sources of information

Participants were further queried about the sources from which they obtained information about LSD before their first-time use. Among the 3136 participants who answered this question, the most commonly cited sources were online forums and websites (59.3%), followed by friends (55.1%). Notably, healthcare professionals and treatment services were among the less frequently utilised sources. Responses for other information sources are illustrated in Table 8.

**Table 8.** Sources of information about LSD accessed by people using LSD for the first time.

Information source	*N* (%)^a^
Online drug forums from the clearnet	1859 (59.0)
Friends	1729 (55.1)
Other websites in the clearnet, e.g. Google	1679 (53.5)
YouTube	1341 (42.8)
Articles published in scientific journals	985 (31.4)
Pill report websites in the clearnet	619 (19.7)
Mass media/news	417 (13.3)
Drug checking/testing/outreach services	367 (11.7)
Social media apps	306 (9.8)
Dealer/vendor (not from darknet)	245 (7.8)
Health care professionals	245 (7.8)
Online drug forums in the darknet	236 (7.5)
Darknet dealer/vendor	88 (2.8)
Other information source not mentioned	314 (10.0)

a Participants could select more than one option, hence percentages totalling greater than 100.

### Expectations and experiences

**Figure 3.**
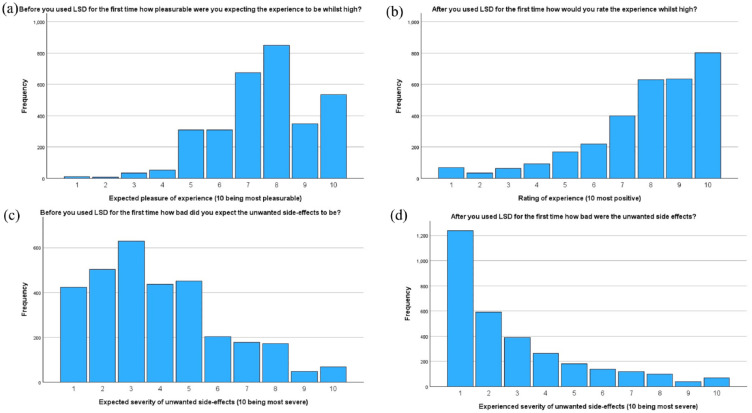
Participant’s expectations and experiences of using LSD. (a) Before you used LSD for the first time, how pleasurable were you expecting the experience to be while high? (b) After you used LSD for the first time, how would you rate the experience while high? (c) Before you used LSD for the first time, how bad did you expect the unwanted side effects to be? (d) After you used LSD for the first time, how bad did you expect the unwanted side effects to be?

Participants’ expectations and experiences regarding the pleasurable effects and unwanted side effects of LSD were also assessed. Prior to their first-time use, respondents reported relatively high expectations of pleasure during the experience, with a mean score of 7.6 (out of 10, 10 being most positive) and standard deviation of 1.7. However, after experiencing LSD for the first time, respondents rated their actual high experiences with a slightly higher mean score of 7.8 and standard deviation of 2.1, indicating generally positive experiences. Regarding the expected severity of unwanted side effects before first-time use, respondents reported a mean score of 3.9 and standard deviation of 2.3 (out of 10, 10 being most severe). However, after the experience, the actual severity of unwanted side effects was rated lower, with a mean score of 2.9 and standard deviation of 2.3. Graphs illustrating the distribution of both positive and negative expectations and experiences are shown in Figure 3.

### Harm-reduction strategies

In terms of harm-reduction practices, participants were asked whether they had taken a small test dose of LSD before their first-time use. Less than a third (31.3%) of respondents indicated that they had taken a test dose, while the majority (67.5%) reported not doing so. A small proportion (1.2%) could not remember whether they had taken a test dose or not.

### Characteristics of EMT-seekers

Participants were asked if they sought EMT after using LSD for the first time. Of the 3132 participants who answered, only a small percentage (0.5%) reported seeking such treatment, suggesting that adverse effects requiring emergency intervention were infrequent among the study participants.

Characteristics of EMT-seekers were explored. Unsurprisingly, EMT-seekers reported a less positive experience (Mann-Whitney U: 17531.5, *Z* = −2.409, *p* = 0.016) and more severe adverse effects of LSD (Mann-Whitney U: 7520, *Z* = −5.277, *p* *<* 0.001) than non-EMT-seekers. Moreover, EMT-seekers reported an average lower mood before taking LSD (Mann-Whitney U: 15307.500, *Z* = −2.950, *p* = 0.003) (see Table 9).

**Table 9.** Characteristics of EMT and non-EMT-seekers.

	EMT-seekers	Non-EMT-seekers	Significance
On the day that you took LSD for the first time, how would you rate your mood before you took it?^a^			
*N*	17	3034	
Median (IQR)	5.0 (3.0–8.5)	8.0 (7.0–9.0)	*p* = 0.003^c^
Before you used LSD for the first time, how pleasurable were you expecting the experience to be?^a^			
*N*	17	3102	
Median (IQR)	8.0 (6.0–9.0)	8.0 (7.0–9.0)	*p* = 0.783^c^
Before you used LSD for the first time, how bad were you expecting the unwanted side effects to be?^b^			
*N*	17	3081	
Median (IQR)	4.0 (3.0–5.0)	4.0 (2.0–5.0)	*p* = 0.508^c^
After you used LSD for the first time, how would you rate the experience while high?^a^			
*N*	17	3088	
Median (IQR)	5.0 (2.0–9.0)	8.0 (7.0–10.0)	*p* = 0.016^c^
After you used LSD for the first time, how bad were the unwanted side effects?^b^			
*N*	17	3095	
Median (IQR)	9.0 (5.5–10)	2.0 (1.0–4.0)	*p* *<* 0.001^c^

a 1–10, 10 being most positive.

b 1–10, 10 being most severe.

c *p*-value for comparisons using Mann-Whitney U Test.

**Figure 4.**
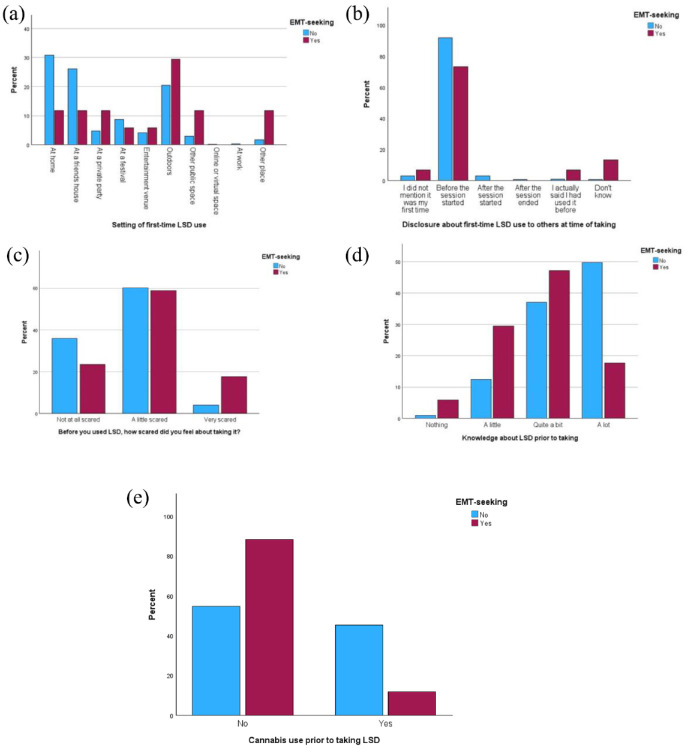
Comparative characteristics of EMT-seekers and non-EMT-seekers. (a) Setting of first-time LSD use. (b) Disclosure about first-time LSD use to others at time of taking. (c) Before you used LSD, how scared did you feel about taking it? (d) Knowledge about LSD prior to taking. (e) Cannabis use prior to taking LSD.

Significant associations were found between EMT-seeking and the setting in which LSD was taken (Fisher’s Exact, *p* = 0.016), whether respondents had told the people that they were with it was their first time (Fisher’s Exact, *p* = 0.004), how scared respondents felt prior to taking LSD (*χ*^2^ = 8.504, *df* = 2, *p* = 0.014), use of cannabis prior to taking LSD (*χ*^2^ = 7.692, *df* = 1, *p* = 0.006), and knowledge of LSD prior to taking (Fisher’s Exact, *p* = 0.006). Characteristics of EMT-seekers are further illustrated in Figure 4.

### Characteristics of people not planning to take LSD again

Participants were asked if they planned to take LSD again. Of the 3135 who answered this question, 40.0% said that they planned to take it again, 43.1% said that they had already taken it again after their first time, 12.3% were unsure whether they would take it again, and 4.6% said that they would not take it again.

In order to determine what factors may have influenced the decision of participants not to take LSD again, the characteristics of participants who said that they would not repeat their LSD use (‘single-timers’) were compared with those who said that they would take or who already had already taken LSD again (‘repeaters’). Summary data on the positive ratings of the experience and the severity of adverse effects under the influence of LSD for both groups are shown in Table 10.

**Table 10.** Characteristics of EMT and non-EMT-seekers.

	Single-timers	Repeaters	Significance
On the day that you took LSD for the first time, how would you rate your mood before you took it?^a^			
*N*	140	2539	
Median (IQR)	8.0 (6.0–8.0)	8.0 (7.0–9.0)	*p* < 0.001^c^
Before you used LSD for the first time, how pleasurable were you expecting the experience to be?^a^			
*N*	142	2597	
Median (IQR)	8.0 (6.0–8.25)	8.0 (7.0–9.0)	*p* = 0.123^c^
Before you used LSD for the first time, how bad were you expecting the unwanted side effects to be?^b^			
*N*	140	2584	
Median (IQR)	4.00 (3.0–6.0)	3.0 (2.0–5.0)	*p* = 0.016^c^
After you used LSD for the first time, how would you rate the experience while high?^a^			
*N*	144	2586	
Median (IQR)	7.00 (4.0–9.0)	9.0 (7.0–10.0)	*p* < 0.001^c^
After you used LSD for the first time, how bad were the unwanted side effects?^b^			
*N*	142	2591	
Median (IQR)	4.5 (2.0–8.0)	2.0 (1.0–4.0)	*p* < 0.001^c^

a 1–10, 10 being most positive.

b 1–10, 10 being most severe.

c *p*-value for comparisons using Mann-Whitney U Test.

Overall, single-timers reported a less positive experience (Mann-Whitney U = 111453.5, *Z* = −8.288, *p* *<* 0.001) and more severe adverse effects (Mann-Whitney U = 110173.5, *Z* = −8.383, *p* *<* 0.001). Additionally, single-timers showed a greater expectation of unwanted side effects prior to taking LSD (Mann-Whitney U = 159188.5, *Z* = −2.420, *p* = 0.016) and a lower mood before taking it (Mann-Whitney U = 148105, *Z* = −3.391, *p* *<* 0.001) (Table 10).

**Figure 5.**
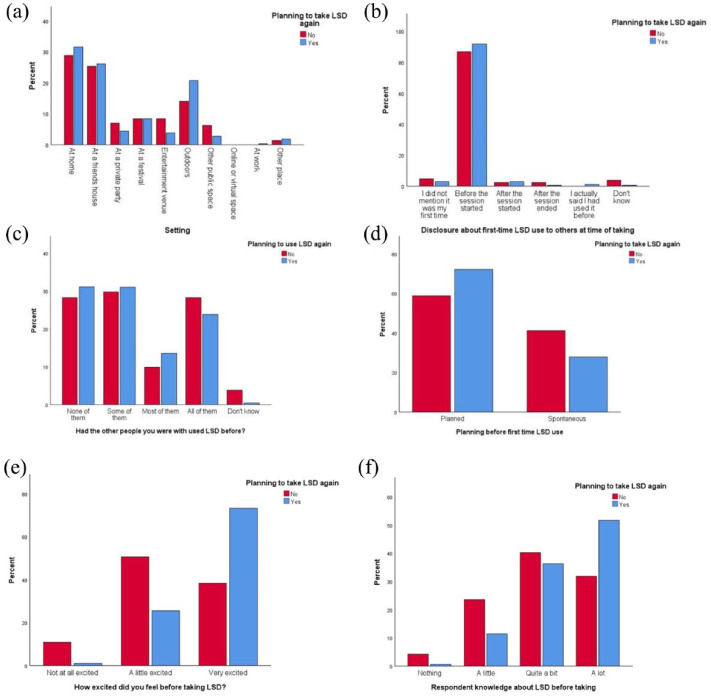
Characteristics of respondents not planning to take LSD again. (a) Setting. (b) Disclosure about first-time use to others at time of taking. (c) Had other people you were with used LSD before? (d) Planning before first-time LSD use. (e) How excited did you feel before taking LSD? (f) Respondent’s knowledge about LSD before taking.

Significant associations were found between the decision to take or not take LSD again and the setting in which LSD was first taken (Fisher’s Exact, *p* = 0.035), whether respondents had told the people that they were with it was their first time taking LSD (Fisher’s Exact, *p* = 0.003), whether the people that they were with had also taken LSD for the first time (*χ*^2^ = 24.267, *df* = 4, *p* *<* 0.001), whether respondents had planned to take LSD or had taken it spontaneously (*χ*^2^ = 12.059, *df* = 1, *p* < 0.001), how excited respondents reported feeling prior to taking LSD (*χ*^2^ = 133.166, *df* = 2, *p* *<* 0.001), and their knowledge of the drug prior to taking (Fisher’s Exact, *p* < 0.001). Characteristics of single-timers are further illustrated in Figure 5.

## Discussion

This study presented comprehensive findings derived from a vast international sample of respondents who participated in the GDS, providing valuable insights into the experiences and perceptions of individuals who engaged in LSD use for the first time within the 12 months before taking the survey. It is the largest study of its kind, reporting data from over 3000 people. The results indicate that the initial experiences of respondents with LSD were predominantly positive, surpassing their preconceived expectations in terms of both pleasure and the limited occurrence of adverse effects. Notably, a significant majority (83.4%) of participants who had used LSD for the first time in the past year expressed their intention to use it again or had already done so. However, the 4.6% of respondents who did not plan to take LSD again (‘single-timers’) reported more negative experiences with the drug. Despite the generally favourable outcomes, a small proportion of respondents (0.5%) sought EMT after their first-time LSD use. This percentage aligns with GDS data from the preceding year, indicating a comparable rate of individuals seeking EMT following LSD consumption. Notably, both EMT-seekers and single-timers differed significantly when compared to other respondents in their responses to questions that asked about setting, disclosure of first-time status to others, mood, and prior knowledge about LSD. Overall, the findings highlight the varying degrees of knowledge about LSD among people using it for the first time and the importance of utilising reliable and accurate sources of information. Moreover, the relatively low utilisation of harm-reduction practices, such as taking a test dose, indicates the potential for increasing awareness and promoting safer practices to reduce potential risks associated with LSD consumption.

While the label of a ‘first-timer’ is suggestive of a novice who lacks knowledge about the drug they are taking, the survey respondents largely seemed to feel well-informed before their first-time use: 37.3% said they knew ‘quite a bit’ about LSD before taking it, and an additional 49.2% knew ‘a lot’ about it. Conversely, among both EMT-seekers and single-timers, there were higher proportions of people who reported knowing ‘nothing’ or ‘a little’ about the drug compared to other respondents. It is possible that this lack of prior knowledge, and thus lack of mental preparation for LSDs’ powerful psychogenic effects, contributed to the negative experiences of these respondents.

Online resources were most commonly used to gather information about LSD. Online drug forums were the most popular resource, which suggests that people using LSD for the first-time value information about the drug from people with prior experience taking it. Online drug forums tend to provide a supportive environment in which harm-reduction techniques, as well as subjectively experienced effects, are shared (Soussan and Kjellgren, 2014). As such, they may be a preferable information source to people who are generally resolved to taking the drug, as most of our first-time respondents − 57.0% said that they ‘always knew [they would] take it 1 day’, and 69.4% said that their first time was ‘planned’ rather than ‘spontaneous’. However, at the same time, drug forums may be less informative about certain aspects of drug safety than more ‘official’ sources, such as pharmacovigilance websites (Karapetiantz et al., 2018). Interestingly, while harm-reduction practices were prevalent among people using for the first time, most reported taking another drug when they took LSD – despite drug-mixing being frequently discouraged by official drug harm-reduction services. The most commonly used drugs were cannabis and alcohol, in keeping with trends of use of these substances in the wider population (Connolly et al., 2020; Morley et al., 2017). Use of cannabis during a psychedelic trip is, anecdotally, suggested as a practice to promote a positive experience, while not necessarily supported by scientific evidence (Palmer and Maynard, 2022), which may be related to the widespread use of peers and online drug forums as information sources prior to taking LSD. Interestingly, in this sample, cannabis use was less frequent in EMT-seekers than non-EMT-seekers. EMT-seekers, however, reported being more scared before taking LSD, so it could be the case that this lower cannabis use was, in general, related to a more anxious (and therefore cautious) mindset, which itself may have been detrimental to the psychedelic experience.

It is also recognised that personality traits and states of mind prior to use can predict the likelihood of positive or adverse psychedelic experiences (Ko et al., 2023). Openness, optimism, and emotional excitability have all been identified as predictors of more positive experiences on psychedelics, but prior apprehension, preoccupation or psychological distress as predictors of negative experiences (Aday et al., 2021). This was reflected in our findings from EMT-seekers and single-timers: both differed from other people using LSD for the first time in their mood, which was significantly lower on average for both groups. Additionally, EMT-seekers were more fearful prior to taking LSD (76.4% reported feeling ‘a little scared’ or ‘very scared’, compared to 64.1% of non-EMT-seekers), and single-timers generally showed less enthusiasm about the prospect of taking LSD (10.9% were ‘not at all excited’ beforehand, compared to 1.1% of repeaters), and their expectation of unwanted side effects, prior to taking the drug, was significantly higher.

For the overall sample of respondents, the current data reveal a picture of mixed emotional states prior to first-time use: 97.7% of respondents reported feeling ‘a little excited’ or ‘very excited’ – a mindset that would be in keeping with the generally positive experience on LSD that was reported. On the other hand, 64.1% also reported feeling ‘a little scared’ or ‘very scared’. It is possible that this element of apprehension may have motivated the use of harm-reduction practices described above. If this were to diminish in the future, for instance, if public interest in and acceptance of psychedelic therapy continues to grow (Aday et al., 2019; Danias and Appel, 2023), this may counterproductively lead to fewer harm-reduction practices being used in a recreational setting. Therefore, it is important to consider the dissemination and public health messaging surrounding LSD as the burgeoning use of psychedelics continues both in licit and illicit settings.

Previous studies have identified that people using psychedelics for the first time tend to use fewer harm-reduction practices (i.e. practices to reduce the likelihood of a challenging experience while under the influence) (Palmer and Maynard, 2022) than their more experienced counterparts. Nevertheless, the current data suggest that such harm-reduction methods are prevalent among people using for the first time: a familiar, comfortable and relaxed setting, where the individual feels secure, is recognised as being important for minimising the risk of adverse psychological effects (Johnson et al., 2008). Accordingly, 56.3% of respondents reported that they took their first dose of LSD at home or at a friend’s house; in contrast, only 23.6% of EMT-seekers took LSD in one of these familiar settings. Additionally, 29.4% of EMT-seekers took LSD outdoors compared to 20.5% of non-EMT-seekers, a setting that has previously been associated with adverse effects (Ona, 2018). Additionally, 79.6% of all respondents reported taking LSD with a close friend, group of friends, partner or lover. When it was taken with other people, the majority of respondents said there was a person present who had prior experience of using the drug. Respondents frequently let others who were accompanying them know that it was their first time before the session had started – suggesting that they were willing to seek the support of those they were with. A lower proportion of EMT-seekers, however, disclosed their first-time use to others, which may be reflective of lower levels of trust in these people.

Finally, 50.3% of respondents said that during their first-time use, there was a person present whose primary role was to take care of the person taking the drug (e.g. a ‘trip-sitter’). While many people taking LSD for the first time took it with friends, a majority (63.3%) said that the influence of their friends on their decision to take it was ‘not strong at all’. This influence may have taken place because many respondents were already resolved to try the drug. Nevertheless, 60.7% of respondents said it was ‘somewhat’ to ‘very’ important that their friends supported their decision to take LSD. This may, in part, have been due to the people taking LSD for the first time wanting to be with a group of friends, thus ensuring a more secure setting. It may also have been related to acquisition of the drug, as 30.4% reported obtaining it from their friends. Moreover, ‘friends’ were cited as the second most commonly used information source about the drug, aligning with the representation of peers as sources of information for other illicit substances (Piatkowski et al., 2023b; Tighe et al., 2017). Given the importance of peers with lived experience as beacons for harm reduction, further investment may be considered in the context of dissemination of harm-reduction information through psychedelic-consumer communities in hopes of this information finding its way into the hands of more end-users.

## Implications

The implications arising from the presented findings are crucial in the context of the growing illicit use of LSD and other psychedelics. It is important to recognise that as public interest and acceptance of psychedelic therapy continue to grow, the apprehension towards LSD use may diminish. This change could potentially lead to a reduction in harm-reduction practices in recreational settings. Notably, both EMT-seekers and respondents not planning to take LSD again reported a significantly lower average mood before taking LSD than other respondents. Given the influence of prior mindset on the psychedelic experience, it is conceivable that low mood contributed to the adverse outcomes seen in some of these respondents. As news of psychedelic therapy for mental illnesses such as depression continues to enter public awareness, it is conceivable that more people with such illnesses seek to ‘self-medicate’ with substances such as LSD. However, our results suggest that such practices are unwise and that promoting a positive mindset prior to LSD use should form part of a comprehensive harm-reduction strategy. Therefore, it is imperative to consider the dissemination of public health messaging surrounding LSD to ensure continued awareness of harm-reduction practices even as the use of psychedelics expands. One encouraging aspect of the findings is the reliance on peers and friends for harm-reduction advice and information about LSD. Many people using LSD for the first time reported obtaining it from their friends, and peers were cited as one of the most common sources of information about the drug. This highlights the potential of utilising peers as conduits to disseminate harm-reduction information within psychedelic-consumer communities. Online forums have previously been suggested as platforms for sharing harm-reduction information (Tighe et al., 2017), and the current findings align with this approach. Drawing on experiences from other illicit substance consumers, who base their use on guidance from ‘expert’ peers, underscores the significance of peer-driven measures to enhance harm-reduction efforts. As the use of LSD and other psychedelics continues to increase, it is essential to harness the power of peer support networks to promote safer practices and harm reduction. Incorporating harm-reduction information within psychedelic communities and utilising lived experiences to guide others can contribute to a more informed and responsible approach to psychedelic use.

Finally, it should be re-emphasised that, while harm-reduction practices were widespread, in certain cases, only a minority or a small majority of respondents adopted such practices (such as taking a test dose or having a ‘trip-sitter’, respectively); additionally, most respondents engaged in polydrug use, a practice that is conventionally considered harmful. Nevertheless, despite these behaviours, the predominantly positive first-time experience of respondents is a finding that is consistent with LSD’s status as one of the safest illicit drugs (Schlag et al., 2022).

### Limitations

This study has several limitations. The retrospective nature of the survey introduces potential recall and response biases, as participants may be influenced by their drug experiences and personal opinions when answering questions about their first-time LSD use. Prospective surveys that capture respondents’ mindset prior to first-time use may yield more reliable insights. Despite this, the study focused on people who had used LSD for the first time recently, ensuring relatively fresh recollections of their experiences. Approximately 57.3% of participants had used LSD within the previous 5 months, enhancing the relevance and reliability of their responses. It is important to note that a considerable portion of participants reported polydrug use on the day of LSD ingestion, which could potentially mediate some effects of LSD. While the examination of characteristics of EMT-seekers can provide useful insights for harm prevention, it should be noted that only a small sample, just 17 respondents, said they had to seek EMT; thus, any generalisation of the findings within this small group to the wider population should be done with caution. Moreover, the influence of selection bias on the results should be considered, as it is likely that individuals who had a positive first-time experience with LSD would be more willing to take part in the survey than those who had a negative experience, such as EMT-seekers or single-timers.

## Conclusion

The findings indicate that most people using LSD for the first time reported positive experiences that surpassed their initial expectations while also indicating a sense of being well-informed about the drug and adopting harm-reduction practices, particularly in relation to the social setting of consumption. However, it is essential to note that some participants engaged in practices that may potentially increase harm, as indicated by using other recreational drugs alongside LSD. Although harm-reduction efforts were observed, they may not have been consciously intentional for all people using the drug. Nevertheless, despite the frequent practice of behaviours that conventionally might be considered harmful in the context of LSD use, the prevalence of adverse outcomes, such as EMT-seeking, remained low. By understanding and emphasising the importance of harm avoidance practices, harm-reduction campaigns can be more effectively tailored to address the increasing use of LSD and other psychedelics. This will help to minimise the risks of negative experiences for people using these substances for the first time in the future. Overall, the results from this study provide valuable insights into the experiences and behaviours of people using LSD for the first time, which can contribute to the development and enhancement of global harm-reduction initiatives in the context of growing interest and consumption of LSD and similar substances.

## Appendix A

**Table:**

Question
1. Thinking about the first time you used LSD, where did you get it from?
2. How long ago did you use LSD for the first time?
3. Thinking about the first time you used LSD, what best describes the situation you were in?*
4. Thinking about the first time you used LSD, which option best describes who you were with?*
5. Thinking about the first time you used LSD, had the other people you were with used LSD before?*
6. Thinking about the first time you used LSD, did you tell at least one of the other people it was your first time?*
7. Thinking about the first time you used LSD, was there a person whose primary role was to take care of you (e.g. trip-sitter/babysitter)?*
8. On the day you took LSD for the first time, how would you rate your mood before you took it?*
9. Before you used LSD on this first occasion, how scared did you feel about taking it?*
10. Before you used LSD on this first occasion, how excited did you feel about taking it?*
11. Before your first time, how certain were you that you would try LSD?
12. Thinking about the first time you used LSD, how strong was the influence of your friends in your decision to try it?
13. Thinking about the first time you used LSD, how important was it for you that your friends supported your decision to try it?
14. Thinking about the first time you used LSD, was this first occasion planned or spontaneous?*
15. Thinking about the first time you used LSD, had you taken any other drugs that day before you took LSD?*
16. On the day that you used LSD for the first time, did you also try another drug/s for the first time?
17. On the day that you used LSD for the first time, what form of LSD did you use?
18. Thinking about the first time you used LSD, how did you take it?
19. Thinking about the first time you used LSD, were you given any advice about how much to take?
20. Before you used LSD for the first time, how much did you know about the drug, its effects, and its risks?*
21. Thinking about the first time you used LSD, did you take a small test dose?
22. Before you used LSD for the first time, what sources did you obtain this information from?
23. Before you used LSD for the first time, how pleasurable were you expecting the experience to be while high?
24. Before you used LSD for the first time, how bad did you expect the unwanted side effects to be?
25. After you used LSD for the first time, how would you rate the experience while high?
26. After you used LSD for the first time, how bad were the unwanted side effects?
27. After you used LSD for the first time, did you seek emergency medical treatment?
28. Do you plan on taking LSD again?

* Questions that were used when examining characteristics of EMT-seekers and people not planning to take LSD again.
